# Attitudes to prenatal screening among Norwegian citizens: liberality, ambivalence and sensitivity

**DOI:** 10.1186/s12910-018-0319-9

**Published:** 2018-09-18

**Authors:** Morten Magelssen, Berge Solberg, Magne Supphellen, Guttorm Haugen

**Affiliations:** 1 Centre for Medical Ethics, Institute of Health and Society, University of Oslo, Pb. 1130 Blindern, N-0318 Oslo, Norway; 2 Department of Public Health and Nursing, The Norwegian University of Science and Technology (NTNU), Trondheim, Norway; 3 Department of Strategy and Management, Norwegian School of Economics, Bergen, Norway; 4 Department of Fetal Medicine, Division of Obstetrics and Gynecology, Oslo University Hospital and University of Oslo, Oslo, Norway

**Keywords:** Bioethics, Down syndrome, Non-invasive prenatal testing, Prenatal diagnosis, Public attitudes

## Abstract

**Background:**

Norway’s liberal abortion law allows for abortion on social indications, yet access to screening for fetal abnormalities is restricted. Norwegian regulation of, and public discourse about prenatal screening and diagnosis has been exceptional. In this study, we wanted to investigate whether the exceptional regulation is mirrored in public attitudes.

**Method:**

An electronic questionnaire with 11 propositions about prenatal screening and diagnosis was completed by 1617 Norwegian adults (response rate 8.5%).

**Results:**

A majority of respondents supports increased access to prenatal screening with ultrasound (60%) and/or full genome sequencing of fetal DNA (55%) available for all pregnant women. Significant minorities indicate, however, that a public offer of prenatal screening for all pregnant women would signal that people with Down syndrome are unwanted (46%) or could be criticized for contributing to a ‘sorting society’ (48%).

**Conclusions:**

Results indicate deeper ambivalences and a cultural sensitivity to the ethical challenges of prenatal screening and subsequent abortions. The specific diagnosis of Down syndrome and the fear of becoming a ‘sorting society’ which sorts human life due to diagnoses, appear to play prominent roles in citizen deliberations. The low response rate means that a non-response bias cannot be excluded, yet reasons why results are still likely to be of value are discussed.

## Background

Throughout the world, politicians, health professionals and lay people must decide how to respond to the ever-increasing possibilities offered by advances in biotechnology. In the field of prenatal diagnosis, new test technologies come with complex moral, medical, political and practical questions. Interestingly, public debates do not proceed in the same fashion in all Western countries. Whereas in some countries novel and potent prenatal diagnostic tests have been introduced with little controversy and public debate, in other countries the opposite has been the case. Norway is one of the latter countries, where debates about prenatal screening have featured prominently on the public agenda, sometimes being among key issues in elections and giving rise to repeated and extensive media debates [1].

In this paper, by ‘liberal’ regulation and ‘liberalization’ we mean regulation which gives access to the procedures in question, with few restrictions in the form of laws or criteria for access. A ‘restrictive’ regulation, on the other hand, outlaws procedures or restricts access through criteria that patients must fulfil. Norway is one of the few countries in the world that regulates access to prenatal diagnosis/screening through hard law. Only a small proportion of pregnant women will have access to prenatal screening such as the CUB test (combined first trimester ultrasound and blood tests) and the NIPT (non-invasive prenatal testing, where cell-free fetal DNA in maternal blood is analyzed). This is an apparent paradox, for Norway’s abortion practices are in other respects liberal; since 1978, first-trimester abortion has been available on request and second-trimester abortion on social indications. In the Norwegian debate there has been a larger concern about *selective* abortion (ie, abortion due to fetal malformation/chromosomal aberration) and, in particular, the increase in selective abortion for Down syndrome. The concept of ‘the sorting society’ (‘Sorteringssamfunnet’) [1] expresses the fear that present selective abortion and prenatal screening practices already sort human life. The ‘sorting’ criticism entails both a reference to potential discrimination against disabled people as well as a vague reference to eugenic selection practices in Nazi Germany. In addition, the concept also refers to parental virtues that might seem threatened by prenatal screening: When pregnant women are offered screening, it could be argued that they are actually invited to abandon the parental virtue of acceptance of any kind of child. Instead, a judging, evaluating or *sorting* mindset will prevail. Among critics of ‘the sorting society’, a fear is that the increased power that comes with technological advances will only strengthen such tendencies in the future. On the other side of the debate, some advocate publically financed access to CUB and/or NIPT for the entire pregnant population, justified primarily by autonomy, but also by equality of access. Future scenarios, such as NIPT for charting the entire fetal genome, are also sometimes discussed.

### Prenatal diagnosis in Norway

Since 1994 prenatal diagnosis in Norway has been regulated by the Biotechnology Act, supposedly the first law worldwide regulating the use of biotechnology in human medicine [2]. Following vigorous public debate, a new law was passed in 2003, defining prenatal diagnosis as “examination of fetal cells, a fetus or a pregnant woman to obtain information about the genetic constitution of the fetus or to detect or exclude a disease or abnormality of the fetus”.^1^

The indications for prenatal screening are mainly unchanged from those stated in a 1983 Director of Health circular [2]. Maternal age 38 years and above (which involves increased risk of chromosomal abnormalities such as Down syndrome – trisomy 21 – and other trisomies) is a main indication, as is past history of a child with a genetic/chromosomal disorder. In 2001, additional indications were added, including situations where the woman/couple is/are unable to take care of a disabled child, and when a previous ultrasound examination has shown raised risk of a chromosomal disorder. Severe anxiety about the development of the pregnancy can also qualifiy. Thus, only a minority of pregnant women have legal access to prenatal diagnosis. According to the 2016 annual report from the five official prenatal diagnostic centres, about 11% of the pregnant population has a prenatal diagnostic/screening test. The most common indication is maternal age and the most common procedure is the combined ultrasound and blood test (CUB test; see below).

A nuchal translucency (NT) scan at gestational week 11–13 to disclose a possible fetal chromosomal disorder can only be performed according to the indications above. From 2005, the results of the scan are combined with results of maternal blood tests to give a risk estimate for the fetus having a chromosomal abnormality, the CUB test. However, it is not prohibited to perform ultrasound examination during gestational age 11–14 weeks due to *other* indications – thus the *intention* with the examination is crucial to establish its legality. This imprecision – or loophole – has created a demand for ultrasound examinations outside the five official fetal diagnostic centres [3]. A 2014 survey at Oslo University Hospital showed that of all women coming to a routine ultrasound examination at week 17–19, about 60% had had a previous ultrasound examination performed outside the hospital that could be interpreted to be an NT scan [4]. The proportion is likely to be lower in other parts of the country. Approximately 90% of women/couples where a diagnosis of Down syndrome is made, go on to choose abortion^2^ (Denmark: 98%^3^). Because the average maternal age has increased, however, the number of children with Down syndrome born has been stable since 2000.

Non-invasive prenatal tests (NIPT) were approved for use in Norway in 2017, but only in situations where the CUB test has shown increased risk of a chromosomal abnormality. The test is not available outside this specific indication, and it is known that some women therefore go abroad to take it.

In a European and Nordic context, Norway’s policy on prenatal diagnosis is restrictive; this was observed initially in 1997 and is still true more than twenty years later [5]. This is striking considering that our culturally similar neighbor Denmark is one of the most liberal countries worldwide [6].

The aim of the present study is twofold. First, we investigate whether the exceptional regulation of prenatal screening/diagnosis in Norway is grounded in public attitudes and cultural sentiments. Second, we examine whether what appear to be exceptional features of the Norwegian debate are mirrored in public attitudes.

## Methods

The study took place as one of four substudies in the 2017 NOBAS (Norwegian Bioethics Attitude Survey) study. In February 2017, the commercial firm Respons Analyse invited members of their nationally representative web panel via email to complete the NOBAS online questionnaire, which they were told would chart attitudes towards ethical issues. Recruitment was made in several waves until the target number of responses had been achieved. In total, 18,976 adult respondents were invited and 1617 completed the questionnaire, for a response rate of 8.5%. Responses were not registered until all questions had been answered. Responses were weighted so that the responses of demographic groups that were under-represented in the survey (Table 1) were given more weight in the analyses.

**Table 1:** Demographic characteristics of respondents

Characteristic	Unweighted (N, (%))	Weighted (N, (%))
Gender		
Female	811 (50.2)	804 (49.7)
Male	806 (49.8)	813 (50.3)
Age		
18–24	110 (6.8)	193 (11.9)
25–34	233(14.4)	282 (17.5)
35–44	254 (15.7)	289 (17.9)
45–54	308 (19.0)	287 (17.7)
55+	712 (44.0)	567 (35.0)
Level of education		
Primary school	74 (4.6)	74 (4.6)
Upper secondary school	396 (24.5)	430 (26.7)
College/university ≤3 yrs	460 (28.5)	443 (27.5)
College/university > 3 yrs	658 (40.7)	647 (40.2)
Unanswered	18 (1.1)	15 (0.9)
Religious beliefs		
Non-religious	728 (45.0)	753 (46.7)
Christian	710 (43.9)	685 (42.5)
Other religions	26 (1.6)	26 (1.6)
Unanswered	148 (9.2)	149 (9.2)

### Questionnaire

The substudy on prenatal diagnosis first presented respondents with the following introduction to the topic:

Prenatal diagnosis by ultrasound and blood tests in pregnancy weeks 11-13 is used in particular to detect Down syndrome or other chromosomal abnormalities in the fetus. This examination is currently offered to pregnant women with the highest risk, which means especially those over 38 years of age. Some believe that the examination should be offered to all pregnant women.

Respondents were then presented with 11 statements to which they should then respond with a level of agreement or disagreement. Five responses were available for each statement: «Fully agree», «somewhat agree», «neither agree nor disagree», «somewhat disagree» and «fully disagree». «Do not know» was not an option. In the analyses two questions from another substudy, concerning attitudes towards abortion law, were also included.

The questionnarie was developed through thorough discussion among the authors; four lay persons also gave feedback on the questionnaire, and two of these tested the electronic version.

### Statistical analyses

Statistical analyses were performed with IBM SPSS Statistics version 25. Results are presented with descriptive statistics and mean Likert scores on a five-point scale, with «fully disagree» (=1) and «fully agree» (=5) as scale anchors. Multivariate analysis of variance (MANOVA) was used to compare attitudes across demographic segments (age, gender, education, religious beliefs).

## Results

The demographic characteristics of the 1617 respondents are detailed in Table 1.

Table 2 shows the responses to the 11 attitude questions concerning ethical and political issues in prenatal diagnosis. A majority of respondents (60%) support the extension of the current public program of prenatal diagnosis to the entire pregnant population without any co-payment, as well as access to NIPT for sequencing of the fetal genome (55%) should this become available. Two-thirds agree that abortion is acceptable if the fetal condition would place a heavy burden of care on the family, yet only 42% agree that a diagnosis of Down syndrome makes abortion ethically acceptable. Few (7.4%) support a moral duty to abort in case of Down syndrome, and a similar proportion (8.3%) think that prospective parents should not expect extensive assistance from the state in such a case. Concerning pressures and expectations when Down syndrome has been diagnosed in the fetus, 56% believe there is often pressure to choose abortion, whereas 25% believe there is often a pressure to continue the pregnancy. Slightly less than half of respondents agree that an offer of prenatal diagnosis to all pregnant women signals that people with Down syndrome are unwanted (46%) or that society in that case could be criticized for being a «sorting society» (48%).

**Table 2:** Attitudes towards 11 issues in prenatal diagnosis (N, (%))

Question	Fully agree	Somewhat agree	Neither agree nor disagree	Somewhat disagree	Fully disagree	Mean Likert score
Q1. Prenatal diagnosis by ultrasound and blood test in pregnancy week 11–13 should be offered to all pregnant women, without co-payment	661 (41)	302 (19)	212 (13)	214 (13)	228 (14)	3.59
Q2. I think pregnant women whose fetus has been shown to have Down syndrome often experience pressure and expectations that they choose abortion	352 (22)	552 (34)	449 (28)	173 (11)	91 (5.6)	3.56
Q3. It is ethically acceptable to choose abortion because the fetus has Down syndrome	373 (23)	314 (19)	339 (21)	257 (16)	333 (21)	3.08
Q4. An offer of prenatal diagnosis to all pregnant women will send a signal that people with Down syndrome are unwanted in society	317 (20)	426 (26)	244 (15)	286 (18)	344 (21)	3.05
Q5. It is ethically more problematic to choose abortion when the child was initially planned and desired (as it usually is in prenatal testing) than when the pregnancy was unplanned and unwanted	221 (14)	443 (27)	430 (27)	181 (11)	341 (21)	3.01
Q6. A society that offers prenatal diagnosis to all pregnant women, can be criticized for being a sorting society	271 (17)	508 (31)	224 (14)	244 (15)	370 (23)	3.04
Q7. Those whose fetus has been diagnosed with Down syndrome, but still complete the pregnancy, should not expect extensive assistance from the state	54 (3.3)	80 (5.0)	119 (7.4)	241 (15)	1122 (69)	1.58
Q8. When the fetus has Down syndrome, the pregnant woman should have an ethical obligation to choose abortion	62 (3.8)	59 (3.6)	154 (9.5)	133 (8.2)	1209 (75)	1.54
Q9. Abortion is acceptable if the fetus has a disorder that would place a heavy burden of care on the family	637 (39)	431 (27)	282 (17)	133 (8.2)	135 (8.3)	3.81
Q10. I think pregnant women whose fetus has been shown to have Down syndrome often experience pressures and expectations that they complete pregnancy (that is, *not* to choose abortion)	98 (6.1)	311 (19)	659 (41)	351 (22)	198 (12)	2.85
Q11. In the future it might be possible to map all fetal genes through a blood test of the mother in early pregnancy. Consider the claim: If such a blood test becomes available, it should be legal in Norway for all pregnant women	564 (35)	326 (20)	307 (19)	190 (12)	230 (14)	3.50

Four questions preselected as particularly significant were then used to examine the influence of demographic factors. Table 3 shows that attitudes vary significantly according to the gender, educational level and religious views, but not age, of the respondents. Males and atheists/agnostics are more often in support of prenatal diagnosis (Q1 and Q11) and selective abortion for Down syndrome (Q3), and, correspondingly, less often agree with the characterization of a “sorting society” (Q6) than do females and Christians. The group with the highest level of education is more critical of prenatal diagnosis (Q1&11).

**Table 3:** Mean scores across demographic groups and comparisons between groups (MANOVA)

Variables	Q1	Q3	Q6	Q11
18–34	3.61	3.14	3.09	3.50
35–54	3.47	3.10	3.07	3.53
55+	3.64	3.11	2.96	3.43
Gender				
Male	3.88^a^	3.38^a^	2.81	3.73^a^
Female	3.27	2.85	3.24^a^	3.23
Educational levels				
Primary school	3.79	2.68^c^	3.04	3.66
Upper secondary	3.75	3.09	2.98	3.76
≤ 3 yrs. higher ed.	3.70	3.20	3.00	3.52
> 3 yrs. higher ed.	3.34^b^	3.12	3.09	3.25^d^
Religious beliefs				
Christian	3.46^e^	2.83^f^	3.22^g^	3.37^h^
Atheist/agnostic	3.65	3.49	2.79	3.59
Other non-religious	3.64	3.30	2.87	3.64

^a^ = the score is significantly higher than for the opposite gender (*p* < 0.001)

^b^ = the mean in this group is significantly lower than the means in the Upper secondary and ≤ 3 yrs. higher ed. groups (*p* < 0.001). The mean in the Primary school group on Q1 is also higher, but the difference is only marginally significant (*p* = .09)

^c^ = the mean in this group is significantly lower than the mean in the ≤3 yrs. higher ed. group (*p* < 0.01). The mean in the > 3 yrs. higher ed. group on Q3 is also lower, but the difference is only marginally significant (*p* = .09)

^d^ = the mean in this group is significantly lower than the mean in the ≤3 yrs. higher ed. group (*p* < 0.01) and also lower than the mean in the > 3 yrs. higher ed. group (*p* < 0.05)

^e^ = the mean difference between the scores for Christians and Atheist/agnostic is marginally significant (*p* < 0.1)

^f^ = the mean for Christians is significantly lower than the means for the two other groups (*p* < 0.001)

^g^ = the mean for Christians is significantly higher than the means for the two other groups (*p* < 0.001)

^h^ = the mean for Christians is significantly lower than the mean for Atheists/agnostics (*p* < 0.001), and marginally lower than the mean for the Other non-religious group (*p* < 0.1)

In addition, we examined the impact of gender on Q7 and Q8, finding that males significantly more often than females agreed fully or somewhat that in case of a Down syndrome diagnosis, those who turn down selective abortion and give birth should not expect state assistance (12.9% vs. 3.6%), or have an obligation to choose abortion (10.3% vs. 4.6%; both differences significant at *p* < 0.001 by chi-square testing).

Table 4 shows that a large majority (85%) support current abortion law. Slightly less than a third (31%) would support an extension of the present time limit for abortion on request from 12 to 16 weeks.

**Table 4:** Attitudes towards abortion law (N, (%))

Question	Fully agree	Somewhat agree	Neither agree nor disagree	Somewhat disagree	Fully disagree	Mean Likert score
QA1. In the first 12 weeks of pregnancy abortion should be available on request	1190 (74)	171 (11)	113 (7.0)	73 (4.5)	71 (4.4)	4.45
QA2. The time limit for abortion on request should be raised to 16 weeks	286 (18)	217 (13)	405 (25)	254 (16)	455 (28)	2.77

## Discussion

The survey presented a series of claims, representing arguments in the debate on prenatal diagnosis, that people may agree or disagree with. By exposing respondents to such articulated arguments, one can discover which arguments have a strong standing in Norwegian culture and which are more marginal. In sum, this gives us a portrait of the public’s attitudes and thinking on this topic. Interpretations are, however, conditional on the validity of the findings despite the low response rate; this is discussed in the limitations section.

The first key question, Q1, confirms that a majority support that pregnant women should be given more test choices, through a liberalization of prenatal diagnosis. A paradox seems to arise with Q3 and the question whether it is acceptable to terminate a pregnancy where the fetus has Down syndrome. Fewer agree to this claim. Thus, even though 60% believe that everyone should be offered a CUB test (which is a risk assessment for Down syndrome; Q1), only 42% think it is ethically justified to act upon a ‘bad’ test result (Q3; in a 2010 study the proportion was 41%) [7]. This apparent paradox disappears when we take into account that we cannot know for sure why the majority supports CUB screening. The test can be justified in numerous ways, e.g., as a test for Down syndrome, a test for more serious anomalies (such as trisomy 18 and 13), a test for early detection and follow-up of twin pregnancies, for early bonding between mother and child and for assurance that all is well [8]. Saying yes to the test does not necessarily commit you to defend termination for Down syndrome.

Comparison with other countries is not straightforward as the few in-depth studies published have typically used different questions and formats. A Danish study from 2009-10 found that 65% supported publically funded prenatal diagnosis for all, whereas 31% would reserve the offer for groups at heightened risk only (as per the present Norwegian system) [9]. Only 2% rejected public financing, and apparently no respondents wanted prenatal diagnosis to become illegal. Our study did not examine further what system the 27% who reject publically funded prenatal diagnosis for all would prefer. That women and Christians were more likely to be opposed to prenatal diagnosis was also found in the Danish study. There, younger respondents were more in favour of prenatal diagnosis; in our study age did not predict attitudes.

Q4–6 investigate what disability scholars like Adrienne Asch [10] has called “the any-particular thesis”. Here we ask whether it is more problematic to abort ‘any-kind-of-child’ (abortion in an unwanted pregnancy) than a ‘particular child’ (abortion in a planned and wanted pregnancy because a particular fetal anomaly is discovered). The results suggest that Ash’s thesis is an apt characterization of Norwegian attitudes: 41% of respondents think it is ethically more problematic to abort ‘a particular child’ (a child that was planned and wanted, and now has an anomaly) than ‘any child’ (the typical unwanted pregnancy), whereas 32% disagree (in the Danish study referenced above, only 10% agree to a slightly differently worded question [9]). 46% believe that a first trimester screening program sends a signal that people with Down syndrome are unwanted in society – often called *the expressivist thesis*, that is, the screening program *expresses* a negative view on certain people’s life [11, 12]. In addition, as many as 48% agree that a society that screens for Down syndrome could be criticized for being a ‘sorting society’, echoing the extensive use of the term in the Norwegian public debate. Notably, this is also a worry among some of the respondents who support liberalization of prenatal diagnosis.

However, none of these answers can without further ado be read as protests against prenatal diagnosis. You can for instance acknowledge that a screening program expresses an offensive message, and still think that this does not constitute a sufficient reason to abandon or prohibit the program. You can agree that terminations in planned and wanted pregnancies raise greater ethical challenges than terminations in unwanted pregnancies, and still defend women’s right to prenatal diagnosis and selective abortions. Nevertheless, these answers confirm *a cultural sensitivity to the ethical challenges of prenatal diagnosis*, which furthermore is more pronounced among women. A substantial part of respondents perceive abortion due to fetal anomalies in planned pregnancies as ethically different from terminations of unwanted pregnancies. Fifty years ago, the public regarded the latter type – abortion on social indication – as the major ethical challenge. This survey indicates that the Norwegian public today regards the former type – abortions on a genetic or ‘eugenic’ indication – as the major ethical challenge.

Comparing Q3 and Q9 highlights another interesting issue. In Q3, 37% do not think it acceptable to abort because the fetus has Down syndrome (a finding similar to the 2010 study) [7]. In Q9, however, only 16% think it unacceptable to have an abortion if the fetus “has a condition that could give the family a heavy burden of care”. One of the reasons that approximately 90% of pregnancies where Down syndrome is diagnosed end in termination is precisely, we surmise, that the women in question believe that the condition could give the family a too heavy burden of care. In this survey, we do not know whether respondents saw Down syndrome as “a condition that could give the family a heavy burden of care” or not. Yet what seems to be the case is that resistance and ambivalence to prenatal diagnosis and selective abortion decreases when we are addressing *burdens* rather than the *diagnosis*. A reasonable explanation could be that a focus on unwanted burdens makes selective and non-selective abortions more similar. ‘Burdens’ could be understood as a social indication for abortion. A diagnosis like Down syndrome, by contrast, invokes associations of a *person* with Down syndrome, making the issue one of selecting away an identity. It is easier to agree to terminate burdens than to terminate identities. According to Tim Stainton, “once identity-constituting characteristics are introduced, a specific subject is also introduced. We are no longer talking about a fetus, but a particular subject” [13]. Several positive identity-constituting traits are often ascribed to people with Down syndrome in the public debate. While we all want to get rid of burdens, we want identities to be recognized. Termination of a fetus with Down syndrome may become politically controversial because it appears to entail a lack of recognition of certain people’s identity – an expression of oppression based on identity [14].

Q2, 7–8 and 10 assess the perceived actual freedom of choice after prenatal diagnosis. Much has been written on the value of non-directive counselling and whether pregnant women experience a moral pressure to terminate a pregnancy with an affected fetus [15]. Arguably, if cultural norms and social control make it seem practically impossible to give birth to a child likely to have special needs, then having formal freedom of choice is of subordinate value. In this study 56% of respondents believed women pregnant with a Down syndrome fetus will experience (social) pressure to terminate the pregnancy, whilst only 16% of respondents believed these women would not experience the pressure. A small minority, mainly male respondents, believed in an ethical duty to terminate and/or withholding welfare subsidies to parents that deliberately gave birth to a child with Down syndrome. This indicates that there are few people out there actually defending opinions that will lead to a moral pressure to terminate, but there are many people who believe that such a pressure exists. Clearly, however, social pressure can take subtler forms than explicitly avowed belief, including unconscious bias, body language, etc.

These findings should not necessarily be interpreted as a confirmation that a pressure to terminate does exist in Norwegian culture. Alternatively, we could read this result more as an expression of fear of a pressure to terminate. The latter interpretation is supported by the answers to Q10. Here we asked whether there could be a pressure the other way around – a pressure to give birth to a child with Down syndrome. This is an ‘unusual’ question – in the bioethics literature, it is hard to find papers addressing such an issue. If 56% had answered that they believe there is a pressure to abort in the previous question (Q2), we should expect that the same 56% would *not* believe there is a pressure to carry such a baby to term. However, the answers are different: Only 34% believes that there is no such pressure, 25% actually believes that there is such a pressure and as many as 41% do not have a clear opinion. This seems to support our former interpretation: The Norwegian culture is quite *concerned about social pressure* in a situation where there is nevertheless formal freedom of choice. Most people believe that there is a pressure to abort, but a quarter of the respondents believe that there is a pressure to give birth to a child with a Down syndrome diagnosis. The results may seem inconsistent; or they may express the deeper ambivalence of this situation, that maybe one can experience a pressure in both directions at the same time.

One result might seem to deviate from the main interpretations presented so far. In Q11, respondents are asked if a future test capable of mapping the fetal genome should be legal in Norway. Here, only 26% indicate that it should not. Bearing in mind that 37% do not think it acceptable to have an abortion due to Down syndrome, one would expect a higher proportion to be critical of a blood test (NIPT) that could test for thousands of genetic diseases in addition to Down syndrome. To explain this finding, we have to return to our interpretation of Q1. The respondents may think that this test, similar to the ultrasound test, may have many different purposes. To “map all fetal genes” is not an expression that is necessarily linked to abortion, but could, in the minds of some respondents, be associated with prevention or treatment. In addition, we cannot expect the public to have thought through the many complex ethical and practical implications of full sequencing of the fetal genome.

### Limitations

The response rate was low at 8.5%, clearly raising the question of a potential non-response bias wherein survey participants differ significantly from those who declined to participate, making results less representative of the target population. The low response rate means that a significant non-response bias unfortunately cannot be ruled out. However, there are six reasons why such a bias is not necessarily present, so that results are still likely to be valid.

First, the demographic characteristics of the respondents correspond well with the national average, and the answers have been weighted to further increase this match. Second, the results fit well with a previous study on the topic [7]. Third, the results are more or less in line with what those among the authors who have followed the Norwegian debate for decades were expecting, with no genuinely surprising results. Fourth, nonresponse bias has been shown to be less of a problem in attitude surveys, such as the present, than in surveys where respondents are asked to detail their activities [16]. Fifth, the invitation email presented the survey as a «survey on attitudes towards ethical questions»; the nature of the topics surveyed was not disclosed. This might, however, have led to respondents taking more of an interest in ethical questions to be more likely to accept the invitation (thus perhaps decreasing the proportion of indifferent answers compared to the true population average); yet it is unlikely to have led to a selection on respondents with certain views. As Fosnacht et al. point out,

The impact of nonresponse on an estimate depends upon the relationship between the outcome of interest and the decision to participate in the survey. Consequently, if survey participation is not correlated with its content, the answers of responders and non-responders to a survey will not substantially differ [17].

Finally, as low and decreasing response rates are a significant problem in most countries, much work has gone into examining whether low response rates necessarily invalidate survey findings. Indeed, detailed analyses indicate that surveys may very well be representative in spite of very low response rates [16, 18].

In sum, although we have found no positive reason to think that the present study has been affected by a significant non-response bias, the low response rate means such a bias, reducing the validity of the findings, cannot be ruled out.

## Conclusion

Our study indicates that prenatal diagnosis and selective abortion is regarded as ethically challenging among the Norwegian public. In spite of a strong support for the Norwegian abortion law, including abortion on demand, the Norwegian public tends to regard abortion after prenatal diagnosis as something different. The exceptional regulation of prenatal diagnosis in Norway appears in fact to be grounded in public attitudes. The closer you get to the diagnosis of Down syndrome the more controversial it becomes. A majority appears to support first trimester screening for all pregnant women, as well as abortion when a family has to face significant burdens. However, when Down syndrome is addressed specifically, the majority acceptance for abortion appears to recede. This means that in Norway the any-particular-distinction as well as the expressivist theses are part of the social matrix Norwegians act upon. Norwegians appear to believe that prenatal diagnosis and selective abortion represent harder ethical challenges than abortion on social indications. They also appear to believe that these services within the health care systems do send signals – they express degrading views of certain people. Finally, Norwegians appear to think that all these concerns are particularly linked to Down syndrome. In sum, liberality, yet also ambivalence and sensitivity, are keywords characterizing Norwegian public attitudes towards prenatal diagnosis.

This study is a study of attitudes, which are not the same as actions. Even though only 42% in this study believe it ethically acceptable to abort after a diagnosis of Down syndrome, we know that almost all (90%) choose a termination if they find themselves in this situation. This statistical fact does not make surveys of public attitudes irrelevant. Rather, we now have an indication of the ‘agony of soul’ experienced by pregnant women in Norway faced with these decisions. On the basis of this study we would form the hypothesis that in a culture that discusses and problematizes prenatal screening and harbours ambivalent attitudes, choices might be experienced as harder and more painful than in cultures wherein abortion for disease and disability is considered as less controversial and as part of preventive medicine. Qualitative interview studies are well placed to examine this further [15].

## Abbreviations

CUB test: Combined first trimester ultrasound and blood tests
MANOVA: Multivariate analysis of variance
NIPT: Non-invasive prenatal testing
NOBAS: Norwegian Bioethics Attitude Survey
NT: Nuchal translucency
Q: Question

## Footnotes

1 A translation of the Act is available at https://www.regjeringen.no/globalassets/upload/kilde/hod/red/2005/0081/ddd/pdfv/242718-biotechnology_act_master.pdf (accessed 30.8.2018)

2 These are the official Norwegian statistics, available at https://www.fhi.no/nyheter/2016/nye-tall-om-down-syndrom-i-norge/ (accessed 30.8.2018)

3 https://www.dr.dk/nyheder/indland/om-30-aar-er-downs-syndrom-udryddet (accessed 30.8.2018)

## References

[1] Melhuus M (2012). Problems of conception: issues of law, biotechnology, individuals and kinship.

[2] Berg K (1997). Prenatal diagnostics in Norway. Eur J Hum Genet.

[3] Røe K, Salvesen K, Eggebø T (2012). Are the Norwegian guidelines for ultrasound in prenatal diagnosis followed?. Tidsskr Nor Legeforen.

[4] Helsedirektoratet. Evaluering av bioteknologiloven 2015. https://helsedirektoratet.no/Lists/Publikasjoner/Attachments/997/Evaluering-av-bioteknologiloven-2015-IS-2360.pdf. Accessed April 30, 2018.

[5] Leschot N, Vejerslev L (1997). Proceedings of the EUCROMIC workshop on prenatal diagnosis. Eur J Hum Genet.

[6] Solberg Berge (2018). From Prenatal Diagnosis to Preterm Infants: A Cultural Guide to Understand Scandinavian Variation. Pediatrics.

[7] Helsedirektoratet. Bioteknologiloven. Undersøkelse om holdninger til etiske problemstillinger. 2010. https://helsedirektoratet.no/Documents/Bioteknologi/bioteknologi-etikk%20undersokelse%20bioteknologiloven%20august2010%20Perduco.pdf. Accessed April 30, 2018.

[8] Renner I (2006). Experience of Pregnancy and Prenatal Diagnosis.

[9] Uldall SW, Norup MS (2013). Attitudes among Danes toward prenatal testing and termination of pregnancy. Acta Obstet Gynecol Scand.

[10] Asch A, Parens E, Asch A (2000). Why I haven’t changed my mind on prenatal diagnosis. Prenatal testing and disability rights.

[11] Hofmann B (2017). 'You are inferior!' revisiting the expressivist argument. Bioethics.

[12] Magelssen M, Materstvedt LJ (2013). Å granske hjerter og nyrer: Ultralydens etikk. Nytt Norsk Tidsskrift.

[13] Stainton T (2003). Identity, difference and the ethical politics of prenatal testing. J Intellect Disabil Res.

[14] Solberg B, Kristiansen K, Vehmas S, Shakespeare T (2010). Prenatal screening for Down syndrome – why we shouldn’t. Arguing about disability: philosophical perspectives.

[15] Risøy S. Sårbar, suveren og ansvarlig. Kvinners fortellinger om fosterdiagnostikk og selektiv abort. PhD thesis. Bergen: University of Bergen; 2010.

[16] Hellevik O (2016). Extreme nonresponse and response bias. Quality Quantity.

[17] Fosnacht K, Sarraf S, Howe E (2017). How important are high response rates for college surveys?. Rev High Educ.

[18] Groves RM, Peytcheva E (2008). The impact of nonresponse rates on nonresponse bias: a meta-analysis. Public Opin Q.

